# Evaluation of long-range temporal correlations during overt and covert attention in a steady state visual evoked potential based brain-computer interface

**DOI:** 10.1371/journal.pone.0345793

**Published:** 2026-04-10

**Authors:** Zafer İşcan

**Affiliations:** Department of Biomedical Engineering, School of Engineering and Natural Sciences, Istanbul Medipol University, Istanbul, Turkey; Wadia Institute of Himalayan Geology, INDIA

## Abstract

Gaze control is required for successful brain-computer interface (BCI) operation in different paradigms. It has been shown that the performance of a steady-state visual evoked potential-based BCI is lower in covert attention when the participants attend to the stimuli covertly, without the need to move their eyes. Some studies in the literature have tried to find the brain regions that are affected by covert attention. Moreover, it has been shown that the signal-to-noise (SNR) ratio is smaller in covert attention than in overt attention. Based on the fact that brain oscillations exhibit long-range temporal correlations (LRTCs), which can be measured by the Hurst exponent, and estimated using the detrended fluctuation analysis (DFA), this is the first study focusing on the DFA differences in overt and covert attention in an SSVEP-based BCI experiment. The main hypothesis is that there should be differences between DFA exponents of EEG in overt and covert attention, as there are differences in SNR between these attentional states. Gender differences between overt and covert attention were also evaluated using DFA. The results revealed significant differences in LRTCs depending on the gender and the attentional state. These results could be taken into account for an efficient SSVEP-based BCI design.

## Introduction

The brain is the most complex organ that orchestrates the functions in the body, and it has the potential to provide control signals for the interaction of people with the outer world by providing a direct communication path through brain-computer interfaces (BCIs) [1].

One caveat of most current BCI systems is their gaze dependency. Users should be able to move their eyes to focus on the presented stimulus and use the BCI efficiently. This requires overt attention. However, BCIs should also work for those who cannot control their gaze. In this situation, a high information transfer rate (ITR) – a common measure to calculate the performance of a BCI – should be reached with covert attention, where the subjects change only the focus of their attention rather than their gaze. The performance of BCI drops drastically in this case [2].

In the majority of the non-invasive BCI systems, EEG data are used as the control signals, and these systems use different paradigms, including motor imagery, event-related potentials, steady-state visual evoked potentials (SSVEPs), auditory steady-state response, slow cortical potentials, and sensorimotor rhythms [3].

SSVEP is one of the most popular BCI paradigms thanks to its robustness and sufficient signal-to-noise ratio (SNR) [4]. SSVEP-based BCIs offer high ITR. However, they also suffer from gaze dependency, as pointed out by Walter et al. [2]. In their experiment, where the participants reacted to sudden luminance changes of the dot arrays (left/ right), they reported that both overt and covert attention conditions increased the SSVEP amplitudes. However, this effect was much stronger in the overt case, which was validated by an attention modulation index. In another study, Kelly et al. [5] replicated their former two-class (i.e., left/ right) BCI experiment based on overt attention using covert attention and obtained approximately a 20% decrease in average accuracy across subjects due to this switch. In their experiment, they used 17 Hz/ 20 Hz as the frequency of the left/ right checkerboard pattern stimuli, respectively, and fast Fourier transform (FFT) based features with a linear discriminant analysis classifier. In another two-class (i.e., left/ right) visual attention task, Ordikhani-Seyedlar et al. [6] analyzed the physiological differences between overt and covert attention. They used paired combinations of four frequencies (6 Hz vs 7 Hz, 8 Hz vs 9 Hz), and they also observed a sharp fall in the power of the SSVEP signal at stimuli frequencies during covert attention compared to overt attention. They reported a consistent increase in the power of the second harmonics of the stimuli frequencies under covert attention. Chen et al. [7] proposed a BCI speller using SSVEP under overt attention and reported a speed (12 words per min.) comparable to eye trackers. They mentioned that such a speller cannot be used with covert attention. Although there were some attempts to increase the classification accuracy of SSVEP-based BCIs under covert attention, the results were not satisfactory. In [8,9] the average online classification accuracies were 72.6 ± 16.1% and 74 ± 13%, respectively, for two-class BCI tasks. These results demonstrate the need for a reliable SSVEP-based BCI that works under covert attention.

Several studies focused on the cortical areas that are responsible for covert attentional shifts. Gunduz et al. [10] mentioned the contribution of fronto-parietal regions for orienting attention using an electrocorticogram. Kulke et al. [11] compared overt and covert attention using EEG with an eye tracker system. They found a greater frontal positivity for covert attention and related this to the inhibition of saccades. In a real-time functional magnetic resonance imaging experiment, Ekenayake et al. [12] reached an accuracy above chance level (25%) in the parietal lobe, lateral occipital cortex, and fusiform face area in a four-class classification problem. In EEG and magnetoencephalogram studies, alpha band power decreased over the hemisphere contralateral to the attended visual field and increased over the ipsilateral hemisphere in the parietal and occipital lobes [13]. Foster et al. [14] showed that the topographic distribution of alpha oscillations can be used to track covert attention both temporally and spatially. Kelly et al. [15] combined SSVEP features based on FFT with parieto-occipital alpha-band modulations and reached an average accuracy of 79% across subjects. However, existing approaches based on SSVEP and alpha activity provide sufficient performance only in binary classification problems [16]. Therefore, there is a need to boost the accuracy in multi-class BCI designs.

Zhou et al. [17] quantified the effect of retinal eccentricity on SSVEP in overt and covert attention. They showed that the participants’ attentional status (overt, covert, or no attention) can be detected with an accuracy of 91.42 ± 5.30%. They also classified different types of covert attention (near, moderate, far) with an accuracy of 66.67 ± 14.21%. It is clear from their study that in the covert attention case, SNR is lower, especially in higher harmonics.

Spontaneous oscillations in the human brain show scaling behavior that can be revealed with scaling analysis [18]. In this behavior, none of the characteristic scales dominate the dynamics of the underlying process, and scaling analysis helps us evaluate the fluctuations of a parameter as a function of the scale [18]. The scale-free behavior exhibits itself as slowly decaying autocorrelations that are called long-range temporal correlations (LRTCs) [19]. A measure of long-term memory of time series is the Hurst exponent, and a Hurst exponent between 0.5 and 1 shows that LRTCs exist in the given data [20]. Detrended fluctuation analysis (DFA) [21] is a common method to estimate the Hurst exponent. In this method, for each selected window size, the linear trend is removed from the cumulative sum of the signal, and the fluctuation is calculated as the mean standard deviation of all identically sized windows. Then the fluctuation values are plotted on logarithmic axes for all window sizes. The slope of the corresponding line in the range of interest gives the DFA exponent (α) [22].

It has been shown that the DFA exponents were attenuated for low SNR (<1) and were marginally affected at higher SNR (>2) values [23]. Samek et al. [24] also found that the Hurst exponent and SNR are positively correlated, and the highest correlations were observed in the alpha band. Their results showed that both Hurst Exponent and SNR are highly correlated with classification accuracy. Pavlov et al. [25] showed that there are differences in DFA exponents among imagined movements, real movements, and background activity, highlighting the potential use of DFA in BCIs. Similarly, Gaurav et al. [26] classified six different cognitive tasks with a high accuracy using a multifractal DFA.

In this study, LRTCs were evaluated in an SSVEP-based BCI experiment during overt and covert attention using DFA exponents. This is the first study focusing on the DFA differences in overt and covert attention in an SSVEP-based BCI paradigm. The study hypothesizes that there should be differences between DFA exponents of EEG in overt and covert attention, as there are differences in SNR between these conditions.

There are gender differences in temporal attentional capture [27]. Inhibition of return (IOR) is a spatial cueing paradigm that manipulates covert attention [28]. In Brown’s experiment [29], women showed higher location-based IOR than men. Ahmadi et al. [30] showed greater fractal dimension and complexity in females than in males for delta, alpha, and beta bands in an eyes-closed resting state experiment. They also found a higher laterality in females in terms of complexity. In another eyes-closed resting-state experiment, Nikulin and Brismar [31] showed that alpha and beta band scaling exponents in females are lower than in males. Therefore, in this study, gender differences were also explored using DFA exponents of EEG in overt and covert attention.

## Materials and methods

### Dataset

The EEG dataset used in this study was collected from 20 healthy students at Istanbul Technical University (10 males with a mean age of 25.2 ± 3.4 years, and 10 females with a mean age of 24.6 ± 2.8 years) from 6 May 2010–1 July 2010 [32]. They gave their written consent before participating in the experiment. The participants didn’t have a neurological disease background. Ethical approval was given by the Faculty of Medicine at Istanbul University.

The original experiment was designed to classify EEG in an SSVEP-based BCI. Four circles were presented to the participants on a laptop screen with individual flickering frequencies (Top: 4.6 Hz, Bottom: 6.43 Hz, Right: 8.03 Hz, Left: 10.7 Hz). The EEG were collected with a 16-channel V-Amp16 system (Brain Products) with active electrodes and digitized with a sampling frequency equal to 500 Hz. The channel locations were TP9, CP5, CP1, CP2, CP6, TP10, P7, P3, Pz, P4, P8, PO9, O1, Oz, O2, and PO10. Ten experimental tasks were summarized in Table 1, each lasting 30 seconds.

**Table 1:** Tasks in the experiment.

Task	Gaze is on	Attention is on
1) Spontaneous, no stimuli	the fixation dot	the fixation dot
2) Attending to the top circle	the fixation dot	the top circle
3) Attending to the bottom circle	the fixation dot	the bottom circle
4) Attending to the right circle	the fixation dot	the right circle
5) Attending to the left circle	the fixation dot	the left circle
6) Focusing on the top circle	the top circle	the top circle
7) Focusing on the bottom circle	the bottom circle	the bottom circle
8) Focusing on the right circle	the right circle	the right circle
9) Focusing on the left circle	the left circle	the left circle
10) Spontaneous, with stimuli	the fixation dot	the fixation dot

In Table 1, tasks #2 - #5 represent the covert attention case where the participants focus their eyes on the fixation point but attend to a specific circle. Tasks #6 - #9 correspond to overt attention, where the participants focus both their eyes and attention on a specific circle. The order of the tasks was according to the table, except for one participant (#12), where tasks #2 and #3 were repeated after task #5. In [32], the authors didn’t use data related to covert attention. In this study, all data in the ten specified tasks are analyzed. For further details regarding the experiment setup and the dataset, please see [32].

### Preprocessing

Although SSVEP is known to be robust against the artifacts in the low-frequency ranges [33], independent component analysis (ICA) was implemented using FastICA 2.5 algorithm (https://research.ics.aalto.fi/ica/fastica/index.shtml) to remove the artifacts. First of all, DC offset was removed from the data. Afterwards, a 4^th^ order, zero-phase Butterworth high-pass filter with a cut-off frequency of 1 Hz was applied before ICA training. To have reproducible and objective artifact removal process, in the estimated independent components (ICs), Welch’s power spectral density estimate was calculated for low (0.5 Hz – 4 Hz), medium (8 Hz – 30 Hz), and high (30 Hz – 80 Hz) frequency bands. Then, the low and high frequency band power ratios were calculated by dividing the low and high frequency power bands by the merged (i.e., low + medium + high) band power. If the low frequency band power ratio was > 0.45, this was determined as a possible blink IC. If the high frequency band power ratio was > 0.4, this was determined as a possible EMG artifact. If the kurtosis was high (> 10), this was saved as a potential artifact. ICs with a low (< 1.2) spatial entropy were also determined as a potential localized artifact. As a result, an artifactual IC was determined by combining these possible ICs. If a possible blink IC had a high kurtosis, this was determined as a blink IC. Similarly, if a possible EMG artifact had a high kurtosis, or a low spatial entropy, this was determined as an EMG artifact. Before applying ICA weights, DC offset was removed from the data and a 4^th^ order zero-phase Butterworth band-pass filter (0.5 Hz – 40 Hz) was applied to the data. After applying the ICA weights, artifact ICs were removed from the data. Then, preprocessed (clean) data was reconstructed using the estimated mixing matrix. As stated by [34], neck strain, swallowing, jaw clenching, and wincing are the most disruptive artifacts for SSVEP. The preprocessing and following data analyses were performed in MATLAB R2023b (MathWorks, Natick MA, USA).

### Long-range temporal correlations

**Fig 1:**
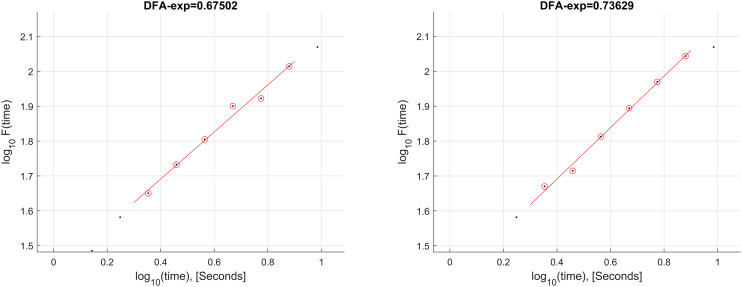
The calculation and fitting interval were given to illustrate the DFA calculation from the Oz channel of a male (left) and a female (right) participant for the task: Spontaneous, no stimuli, delta band.

LRTCs were estimated with DFA using the Neurophysiological Biomarker Toolbox [22]. First, the signal's amplitude envelope was calculated using the absolute Hilbert transform of band-pass filtered data. The signal was band-pass filtered using a finite impulse response filter in five bands: Delta (1–4 Hz), theta (4–8 Hz), alpha (8–12 Hz), beta (12–25 Hz), and gamma (25–40 Hz). The filter window was set to cover at least two periods of the selected band [22]. That means the filter window was equal to 0.25 (2 × 0.125) seconds for the alpha band. The minimum time window was selected to be 2 s, to eliminate filter-induced correlations. The fluctuation function was calculated for time windows from 1 to 10 s with 50% overlapping windows to increase the number of windows in the calculation, and the scaling exponent was obtained by fitting the fluctuation function over the 2–8 s range, excluding very short time scales affected by noise and preprocessing, and longer time scales with insufficient statistical support given the 30 s trial duration. In Fig 1, the calculation and fitting interval were given to illustrate the DFA calculation from the Oz channel of a male (participant #1) and a female (participant #3) participant for the task: Spontaneous, no stimuli, delta band.

### Sex differences

The mean and the median differences between males and females (males – females) were compared with the t-test, and Wilcoxon’s rank sum test, respectively, using the scaling exponents (α) of neuronal LRTCs. The effect size for the difference in mean α values was measured with Hedges’ g due to the small sample size. Both Bonferroni and FDR corrections [35] were implemented due to multiple comparisons, including different frequency bands and experimental tasks (David Groppe (2025) function fdr_bh (https://www.mathworks.com/matlabcentral/fileexchange/27418-fdr_bh), MATLAB Central File Exchange. Retrieved February 13, 2025). Topographical maps for the scaling exponents were generated using eegplot (Ikaro Silva (2024) function eegplot (https://www.mathworks.com/matlabcentral/fileexchange/3279-eegplot), MATLAB Central File Exchange. Retrieved June 6, 2024). Triangle-based cubic interpolation was used to generate the topography for the specified 16 channels.

### Covert-overt attention differences

The mean and the median differences between overt and covert attention across subjects or channels were compared with the t-test, and Wilcoxon’s rank sum test, respectively, using the scaling exponents (α) of neuronal LRTCs using both Bonferroni and FDR corrections [35] due to multiple comparisons, including different frequency bands and paired experimental tasks. There were five paired comparisons: Task 1 (spontaneous, no stimuli) vs Task 10 (spontaneous, with stimuli), Task 2 (attending to the top circle) vs Task 6 (focusing on the top circle), Task 3 (attending to the bottom circle) vs Task 7 (focusing on the bottom circle), Task 4 (attending to the right circle) vs Task 8 (focusing on the right circle), and Task 5 (attending to the left circle) vs Task 9 (focusing on the left circle).

### Band differences

The mean scaling exponents of EEG bands were compared across subjects or channels using ANOVA. Then, multiple comparison tests were used to find the estimates and the confidence intervals of the mean scaling coefficients in different bands.

## Results

### Sex differences

**Fig 2:**
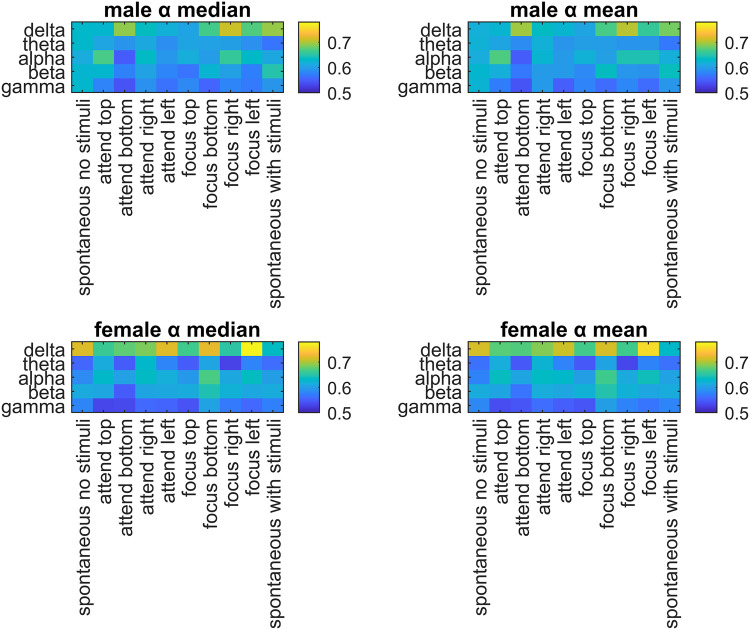
The median and the mean DFA exponents (α) are presented for different EEG bands vs tasks in males and females.

The DFA exponents (α) were calculated for males and females across different EEG bands and experimental tasks (See Fig 2).

**Fig 3:**
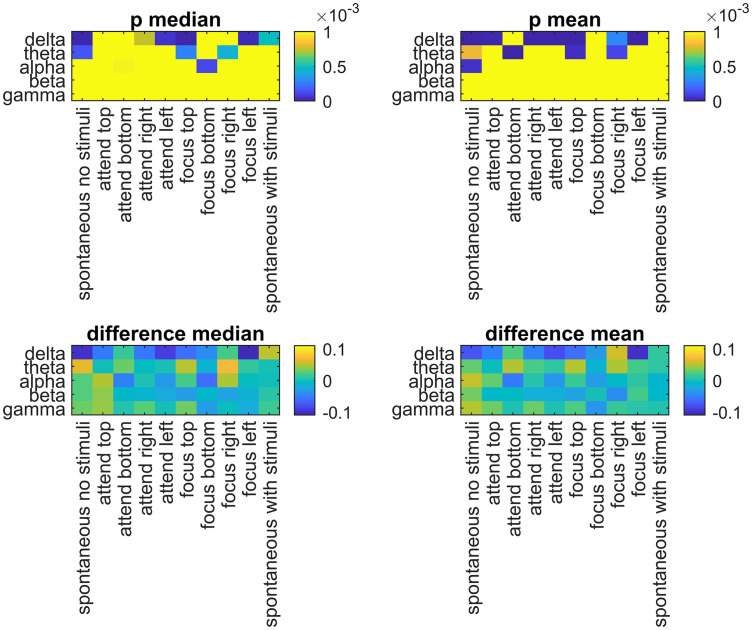
The median and the mean differences in DFA exponents of males and females (males – females) in five different EEG bands and experiment tasks. The yellow areas in the p values show non-significant differences (Using the threshold for Bonferroni correction).

**Fig 4:**
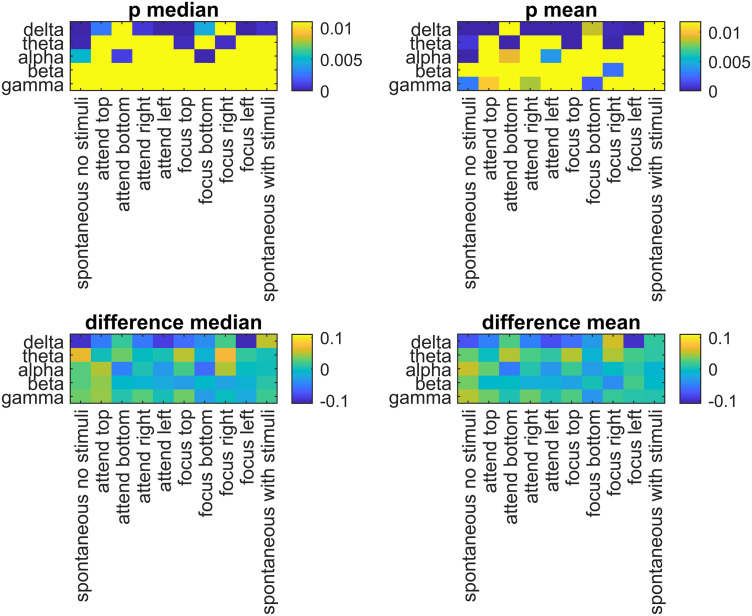
The median and the mean differences in DFA exponents of males and females (males – females) in five different EEG bands and experiment tasks. The yellow areas in the p values show non-significant differences (Using the threshold for FDR correction).

The mean and the median DFA exponent differences between males and females (males – females) were calculated, and the significant differences were marked for Bonferroni and FDR corrections in Figs 3 and 4, respectively, where the non-significant p values were represented with a yellow color.

**Fig 5:**
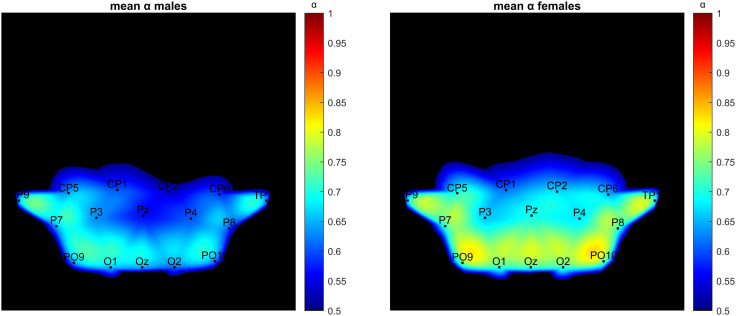
The topographies of the mean α values over the scalp were presented channel-wise for the male (left) and the female (right) participants in the delta band for Task #1 (Hedge’s g = −2.37).

In Fig 5, the topographies of the mean α values over the scalp were presented channel-wise for the male and female participants in the delta band for Task #1.

In Table 2, the effect sizes of these differences were presented in terms of Hedge’s g for all bands & tasks.

**Table 2:** The effect sizes of the differences in the mean α values between males and females (males – females).

task #	1	2	3	4	5	6	7	8	9	10
**delta**	−2.37	−1.96	0.75	−2.09	−3.14	−3.05	−0.81	1.36	−2.69	0.47
**theta**	1.47	−0.09	2.22	0.87	0.52	1.86	−0.36	1.58	0.64	0.42
**alpha**	2.12	0.73	−1.08	0.22	−1.20	0.36	−1.15	−0.15	0.17	−0.31
**beta**	0.92	−0.23	−0.09	−0.58	−0.41	−0.53	0.05	−1.16	0.36	−0.21
**gamma**	1.08	1.09	0.10	1.21	0.06	0.68	−1.25	0.50	0.20	0.13

**Fig 6:**
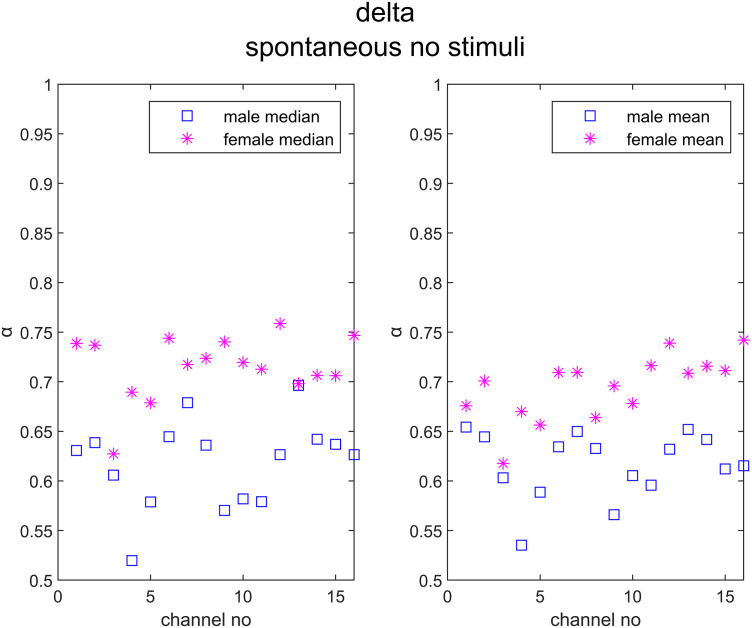
The median and the meanα values for males and females for the delta band in Task #1.

The exact values of the median and the mean α values for males and females were given in Fig 6 for the delta band in Task #1 (i.e., spontaneous, no stimuli).

### Covert-overt attention differences

#### Subject differences averaged across channels.

There was no significant difference between the median DFA exponents of subjects averaged across channels between covert and overt attention. However, there was a significant difference (p < 0.01) between the mean DFA exponents of subjects averaged across channels between covert and overt attention in the beta band for the bottom circle after the Bonferroni correction. The FDR correction didn’t reveal any significant difference.

#### Channel differences averaged across subjects.

**Fig 7:**
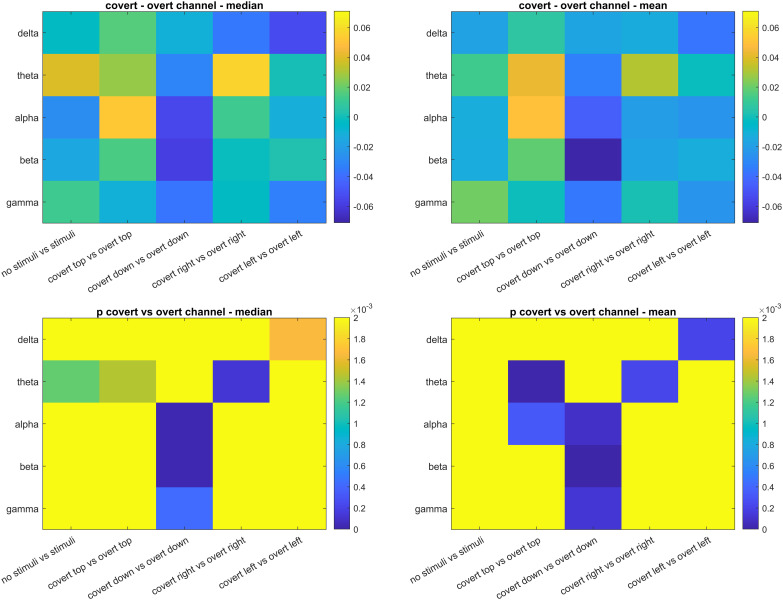
Evaluation of the median and the mean DFA exponents of channels averaged across subjects between covert and overt attention. The yellow areas in the p-values show non-significant differences (Using the threshold for Bonferroni correction).

**Fig 8:**
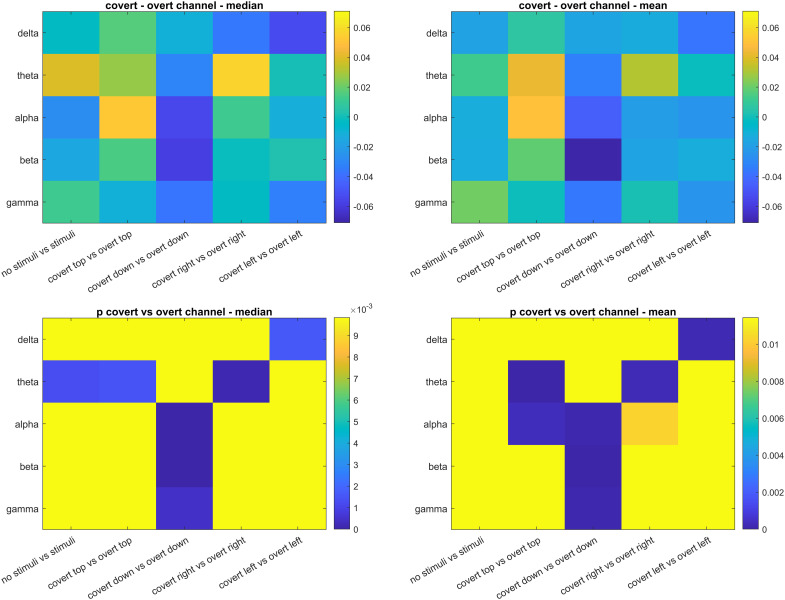
Evaluation of the median and the mean DFA exponents of channels averaged across subjects between covert and overt attention. The yellow areas in the p-values show non-significant differences (Using the threshold for FDR correction).

Significant differences were observed between the median and the mean DFA exponents of channels averaged across subjects between covert and overt attention, as presented for Bonferroni and FDR corrections in Figs 7 and 8, respectively, where the non-significant p-values were represented with a yellow color.

### Band differences

#### Band differences of subjects averaged across channels.

**Fig 9:**
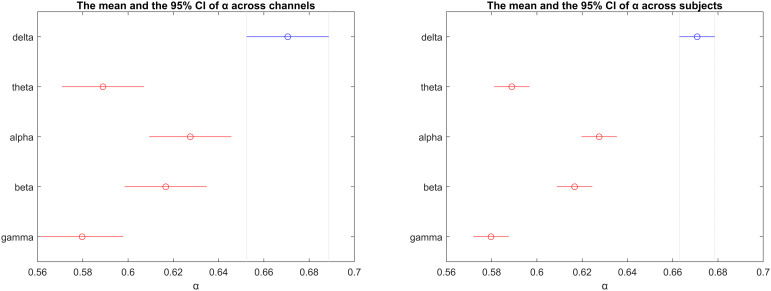
The mean and the 95% confidence intervals of scaling coefficients of subjects across channels (left) vs the mean and the 95% confidence intervals of scaling coefficients of channels across subjects (right).

The mean scaling coefficients of subjects averaged across channels in different bands were significantly (p < 0.001) different. In Fig 9 (left) the mean and the 95% confidence intervals are given to show the spread of the scaling coefficients.

#### Band differences of channels averaged across subjects.

The mean scaling coefficients of channels averaged across subjects in different bands were significantly (p < 0.001) different. In Fig 9 (right) the mean and the 95% confidence intervals are given to show the spread of the scaling coefficients.

## Discussion

In this study, the differences in scaling coefficients (α) during different tasks and EEG bands were evaluated with explorative analyses. Regarding sex differences, in Fig 2, it was shown that female participants consistently had higher mean and median DFA exponents than males in the delta band for many tasks. This result was confirmed with both Bonferroni (Fig 3) and FDR-based (Fig 4) significance maps. In Fig 5, this phenomenon was visualized using the topographies for Task #1 (spontaneous, no stimuli), between the mean α values of males and females in delta band, with a Hedge’s g = −2.37, denoting a very large effect. For this specific band and the task, the mean α values were higher in females than males in all channels (Fig 6). In a previous study by Nikulin and Brismar [31], LRTC analysis focused on alpha and the beta bands in a closed-eye experiment. Therefore, the delta band results in this open-eye study are not comparable with that study. However, Güntekin and Başar [36] showed that brain oscillations are affected by gender differences during visual stimulation. Although they didn’t check LRTCs, they found the largest amplitude difference between the genders in delta band. In Table 2, the effect sizes of the differences in the mean α values between males and females (males – females) were presented for all EEG bands and tasks. The effect sizes were relatively larger in the delta band compared with the other bands in 7 out of 10 tasks. In the theta band, males showed higher mean and median DFA exponents in Task #1, #6 (focus top), and #8 (focus right) (Fig 2) for both Bonferroni and FDR corrections. In the alpha band, females showed higher mean and median DFA exponents in Task #3 (attend bottom) and lower mean and median DFA exponents in Task #1 after FDR correction. For this band, Bonferroni corrected results matched the FDR corrected results in Task #3 and in Task #7 (focus bottom) for the median DFA exponents (Fig 3), and they matched in Task #1 for the mean DFA exponents. For the beta band, the mean and median DFA comparisons didn’t reveal significant differences in Bonferroni corrected results (Fig 3). However, female subjects showed higher mean DFA exponents for the Task #8 (focus right) in the FDR corrected results (Fig 4). In the gamma band, males showed higher mean DFA exponents for the Tasks #1, #2 (attend top), and #4 (attend right) but lower mean DFA exponents for the Task #7 (focus bottom) after FDA correction (Fig 4). These results support that the gender differences in DFA exponents are sensitive to the attentional states. This could be due to the differences in the attended or focused stimulus frequency.

Regarding the differences in the median and the mean DFA exponents of subjects averaged across channels between covert and overt attention, there was a significant difference only in the mean DFA exponents of the beta band for the covert down vs overt down comparison after the Bonferroni correction. The FDR correction didn’t reveal any significant difference.

The median and the mean DFA exponents of channels averaged across subjects between covert and overt attention revealed many significant differences: In the delta band, higher median and mean DFA exponents were observed in overt attention for the left circle with both Bonferroni (Fig 7) and FDR-based (Fig 8) corrections. In the theta band, lower median and mean DFA exponents were observed in overt attention for the top and right circles with both Bonferroni (Fig 7) and FDR-based (Fig 8) significance maps. Besides, both corrections revealed lower median DFA exponents in Task #10 (spontaneous with stimuli) than Task #1 (spontaneous no stimuli) (Figs 7 and 8). In the alpha band, higher median and mean DFA exponents were observed in overt attention for the bottom circle with both Bonferroni (Fig 7) and FDR-based (Fig 8) corrections. For the top circle, lower mean DFA exponents were observed in overt attention with both Bonferroni (Fig 7) and FDR-based (Fig 8) corrections. The mean DFA exponents were higher in overt attention with FDR-based correction (Fig 8) for the right circle. In the beta band, similar to the alpha results, higher median and mean DFA exponents were observed in overt attention for the bottom circle with both Bonferroni (Fig 7) and FDR-based (Fig 8) corrections. In the gamma band, higher mean DFA exponents were observed in overt attention for the bottom circle with both Bonferroni (Fig 7) and FDR-based (Fig 8) corrections. The results demonstrate that there are differences in DFA exponents of EEG channels between covert and overt attention depending on the spatial location. By taking the positive and the negative changes into account, more efficient BCI systems can be designed.

Finally, in Fig 9, different mean α values in different bands showed that there are LRTCs in all EEG bands. However, their ranges differ. The highest mean α values were obtained in the delta band. This could be due to the high SNR value of the delta band [37].

In the study, the record length (30 s) for each task was short. Therefore, the obtained DFA coefficients could be sensitive to the selection of the calculation parameters. Sugimura et al. [19] estimated the reliability of the DFA coefficients obtained from non-overlapping 20 s EEG records using the intraclass correlation coefficient (ICC), and they concluded that the result was acceptable. A recent study proposed the use of Hurst-Kolmogorov method in estimating the Hurst exponent to overcome the poor performance of DFA when the time series is short [38].

Due to the relatively small sample size of 20 subjects, the study has limited statistical power, especially for gender-based conclusions. More subjects will be needed to evaluate the generalization of the results. One caveat of the study is that the order of the tasks was not randomized. Therefore, the results could be affected by the order effects. Besides, there could be some differences in centro-frontal regions between different genders [30] and attentional states. Unfortunately, in the study, it was not possible to evaluate these differences due to the lack of electrodes in these regions. Future studies should randomize the order of the tasks to avoid possible order effects and cover the whole scalp area using more channels (e.g., 32) to have a complete picture of the topographical changes. In the dataset, the eyes were open during the resting state (Task #1). Therefore, the results can not be directly compared with the eyes-closed resting state studies [30,31]. A follow-up study should include the eyes-closed task to enable this comparison. Another limitation was the lack of an eye-tracking system to verify gaze fixations.

SNR or spectral analyses (e.g., FFT) are based on signal amplitude or power at stimulation frequencies. DFA characterizes the temporal organization and LRTCs of neural activity. It captures how neural fluctuations evolve rather than how strong the oscillatory response is at a specific frequency. While SNR in SSVEP reflects attentional gain modulation, DFA provides attention-related differences in neural dynamics and temporal dependency structure. These are not accessible through traditional spectral analyses. The obtained results suggest that overt and covert attention differ not only in response strength but also in the scale-free temporal structure of the underlying neural signals.

The main goal of the study was not to optimize or benchmark a BCI classifier, but rather to investigate attention-related differences in neural dynamics between overt and covert attention tasks using DFA. DFA-derived measures could be complementary features to conventional features. This could potentially improve robustness in some cases where SNR differences are small. The results might also be used for attention state monitoring and/or classification.

The results of the study showed significant differences in LRTCs based on gender and attentional state in an SSVEP-based BCI. These results could help in designing efficient SSVEP-based BCIs in the future by taking the attentional state and gender information into account.
